# Pulmonary sarcoidosis masquerading as metastatic breast cancer: a case report

**DOI:** 10.11604/pamj.2021.38.245.28421

**Published:** 2021-03-08

**Authors:** Amol Dongre, Trupti Dongre, Mahesh Deshmukh, Suraj Agrawal, Niraj Kanchankar

**Affiliations:** 1 Department of Medical Oncology, Alexis Multispecialty Hospital, Nagpur, Maharashtra, India,; 2 Department of Pathology, Narendra Kumar Prasadrao (NKP) Salve Institute of Medical Sciences and Research Centre and Lata Mangeshkar Hospital, Nagpur, Maharashtra, India,; 3 Department of Pathology, Alexis Multispecialty Hospital, Nagpur, Maharashtra, India,; 4 Department of Surgical Oncology, Alexis Multispecialty Hospital, Nagpur, Maharashtra, India,; 5 Department of Radiology, Alexis Multispecialty Hospital, Nagpur, Maharashtra, India

**Keywords:** Sarcoidosis, breast cancer, intraductal carcinoma, non-caseating granuloma, case report

## Abstract

Pulmonary lesions on imaging are presumed to be metastatic lesions in patients with breast cancer. Here, we report an interesting case of a 63-year-old lady with breast carcinoma showing pulmonary lesions on imaging suggestive of pulmonary metastases. Detailed evaluation of pulmonary lesions confirmed the presence of co-existing pulmonary sarcoidosis. Modern diagnostic methods like 18-flurodeoxyglucose positron emission tomography (18-FDG PET) are unable to clearly differentiate metastatic disease from granulomatous diseases like sarcoidosis. Thus, histological confirmation is needed for accurate staging and determining response to treatment and rarely, in non-responders, detecting any co-existing disease. This case emphasizes the need for detailed histopathological examination of lymph nodes in patients with non-responsive disease or recurrent disease despite adequate chemotherapy.

## Introduction

Breast carcinoma in the leading cancer among the Indian female population. Since 1990 to 2016, the age standardised rate of breast cancer has soared by 39.1% with an increase in all the states of the country [1]. The overall 5-year survival rate drops from 99% for localised disease to 27% for metastatic disease [2]. Breast cancer commonly metastasizes to intra-thoracic lymph nodes, but can involve mediastinal or hilar lymph nodes as well [3]. Sarcoidosis is a chronic, multi-system inflammatory disorder of unknown etiology characterized by non-caseating granulomas in lungs as well as draining intra-thoracic lymph nodes [4]. Hilar lymphadenopathy is a common mode of presentation in patients with asymptomatic pulmonary sarcoidosis [5]. The association of breast malignancy with sarcoidosis or even sarcoidosis-like reaction has been rarely reported. Here, we report the case of an elderly lady who was initially managed as a case of breast carcinoma with pulmonary metastasis till further investigations revealed co-existing pulmonary sarcoidosis.

## Patient and observation

**Figure 1:**
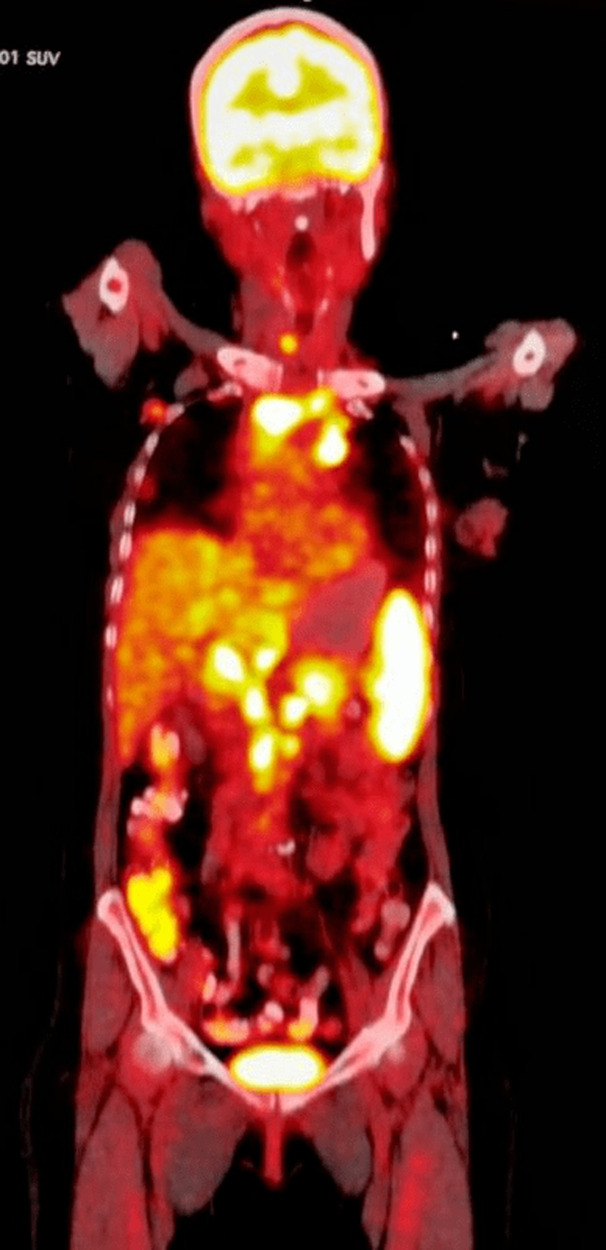
18-FDG PET demonstrating hypermetabolic right axillary nodes, right internal mammary node, mediastinal/hilar, upper abdominal lymphadenopathy along with diffuse splenic hypermetabolism

**Figure 2:**
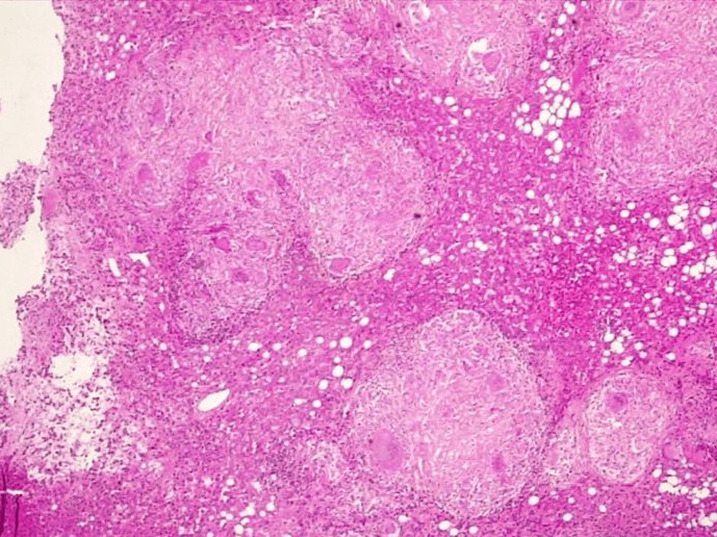
histopathological examination of specimen from EBUS-TBNA showing epithelioid histiocytes and langerhans type of giant cell suggestive of granulomatous inflammation

A 63-year-old elderly female was admitted to our hospital with a lump in her right breast, first noticed two months ago. On clinical examination, a 6x4 cm lump was seen involving the central quadrant, firm to hard in consistency with palpable ipsilateral axillary lymph node. Mammography was suggestive of breast imaging-reporting and data system (BIRADS) category V lesion of the breast. Fine needle aspiration cytology indicated it as ductal malignancy. Furthermore, biopsy confirmed it as grade III intra-ductal carcinoma with ER/PR and HER2-Neu positive malignancy. Eighteen (18)-flurodeoxyglucose positron emission tomography (18-FDG PET) demonstrated a hypermetabolic lobulated soft tissue mass involving outer quadrant of right breast measuring approximately [4.8 x 3.3 x 5 cm SUV max 21.1]. In addition, hypermetabolic right axillary nodes [SUV max 23.8], right internal mammary node [6SUV max 6], mediastinal/hilar and upper abdominal lymphadenopathy [SUV max 17.9] were also seen (Figure 1). Diffuse splenic hypermetabolism was also seen. The patient was planned for neoadjuvant chemotherapy. She received 4 cycles of chemotherapy with AC (Adriamycin and cyclophosphamide) regimen at 3 weeks interval. She tolerated the chemotherapy well. After the fourth cycle, the lump showed a reduction in size to 2 x 2 cm with no palpable axillary lymph nodes. Repeat 18-FDG PET showed near complete metabolic and significant morphologic resolution of previously seen lobulated soft tissue mass involving right breast, now measuring 1.8 cm [SUV max 2.1] and right axillary lymph nodes [SUV max 2.5] was seen. However, there was no gross interval change in size and metabolism of previously seen small supraclavicular, internal mammary, multistation mediastinal/hilar, upper abdominal lymphadenopathy and splenic hypermetabolism. This raised the possibility of an alternate diagnosis. Endobronchial ultrasound guided transbronchial needle aspiration (EBUS-TBNA) was performed to rule out metastasis. Histopathological examination showed epithelioid histiocytes and langerhans type of giant cell suggestive of granulomatous inflammation (Figure 2). No atypical/malignant cells were seen. Interferon gamma release assay (IGRA) for tuberculosis was negative. On further evaluation, angiotensin converting enzyme (ACE) level were found to be elevated (52 IU/L). As she had no pulmonary symptoms, she was not treated for sarcoidosis. Thus, effective management via histopathological examination made sure she did not receive further cycles of unnecessary chemotherapy.

## Discussion

Sarcoidosis is due to a change in the cellular immune response after the immune system is exposed to an infectious, environmental or occupational risk factors [4]. Sarcoidosis has the potential to involve multiple organs including breast tissue. Hence, sarcoidosis may mimic as breast cancer accounting for diagnostic difficulties. The distinction between sarcoidosis involving breast and malignancy of breast is clinically difficult. The differentiation may require radiological and histological investigations when the dilemma remains. Common features among the two diseases imply that malignancy and sarcoidosis may indeed have a similar pathogenesis [6]. Historically, Brincker H and Wilbek E first observed this link in their study on a large cohort (2544) of pulmonary sarcoidosis [7]. They observed that the incidence of lymphoma was 11 times and that of lung cancer was 3 times more common in pulmonary sarcoidosis than general population [7]. Recently, sarcoidosis is being increasingly recognised after cancers such as lymphomas, breast cancer is implicative of a possible causal relation between these malignancies and sarcoidosis [8]. In a study, incidence of sarcoidosis was among patients with breast cancer were 1: 1304 cases, roughly translating to being 38 times more common. In over 50% of these patients, a diagnosis of sarcoidosis was made within 5 years of breast malignancy [8]. The most common pattern of lymph node involvement is intra-thoracic, usually mediastinal or hilar with or without lung parenchymal involvement [5,9]. Definitive diagnosis is established on the basis of a consistent clinic-radiological pattern, collectively with histopathological evidence of non-caseating granulomas [5].

Breast involvement has been infrequently reported in sarcoidosis [10,11]. Co-existing non-caseating granulomas in lymph nodes or organs in individuals with active malignancy is well documented in literature [12]. Surprisingly, there can also be a “sarcoid-like reaction” among individuals with malignancy, found in close proximity to primary tumour site or its draining lymphatics. The exact reason of sarcoid-like reaction is poorly understood. This “sarcoid-like reaction” has been observed to be notably prevalent among patients with testicular cancer and lymphoma, in addition to other various malignancies [13,14]. A definitive diagnosis of sarcoid-like reaction can only be established in the absence of other classical features of sarcoidosis. If sarcoid-like granulomas are identified in patients of breast cancer, a thorough clinical investigation is required to detect the possible presence of sarcoidosis [6]. In our patient, the lymph node involvement was seen in the 18-FDG PET. When the lymph nodal size did not regress after chemotherapy, this raised the suspicion for another underlying pathology. Hence, considering this it may be assumed that sarcoidosis preceded breast malignancy and was only detected when 18-FDG PET study was performed to evaluate spread of breast malignancy. Some authors have described an increased incidence of lymphoma, lung cancer and skin cancers in patients with sarcoidosis [7,13,15] whereas others have not [16,17]. Distinguishing between metastatic and granulomatous lymph nodes can be challenging even when using newer diagnostic modalities with improved discriminatory power such as 18-FDG PET. This makes the histopathological examination exclusive for determining the true nature of the underlying disease. Most common site for this diagnostic dilemma is the involvement of axillary lymph nodes, posing a diagnostic challenge in the differentiating sarcoidosis from suspected metastatic deposits of breast malignancy. The co-relation between sarcoidosis and malignancy remains unclear.

## Conclusion

In conclusion, although the simultaneous presence of sarcoidosis and breast cancer is uncommon, these conditions can co-exist leading to diagnostic difficulties causing dramatic upgrading of breast cancer resulting in treatment with unnecessary chemotherapy. In such cases, histopathological examination is of essence and can help in differentiating among the various etiologies of pulmonary lymphadenopathy, thus, optimising the staging and treatment.
